# Comparing the effectiveness of online and onsite training for simulation facilitator: a quasi-experiment

**DOI:** 10.3389/fmed.2026.1854386

**Published:** 2026-08-13

**Authors:** Lai Kun Tong, Mio Leng Au, Bing Xiang Yang, Ting Li, Yue Yi Li

**Affiliations:** 1 Kiang Wu Nursing College of Macau, Macau, Macao SAR, China; 2 School of Nursing, Wuhan University, Wuhan, China; 3 School of Nursing, Southern Medical University, Guangzhou, China

**Keywords:** core competency, debriefing skills, online training, onsite training, self-efficacy, simulation facilitator training, willingness

## Abstract

**Background:**

With the increasing integration of simulation-based education in healthcare, the role of simulation facilitators has become pivotal. This study aimed to compare the effectiveness of online versus onsite training for simulation facilitators.

**Methods:**

A quasi-experimental design was employed. Participants were assigned to either an online training group or an onsite training group based on their availability and preference, with both groups receiving identical and synchronized training content. Data were collected at baseline and immediately post-intervention.

**Results:**

Of 219 recruited participants, 100 completing the intervention and evaluation (78 online, 22 onsite). Both groups improved significantly in core competencies, with no significant difference in the percentage change between the groups. The online training group demonstrated significant enhancements in debriefing skills and simulation-based education self-efficacy, whereas the onsite group showed improvements that did not reach statistical significance. However, adjusted analyses revealed no statistically significant between-group differences in the percentage change from baseline for simulation facilitator core competency, debriefing skills, simulation-based education self-efficacy, or willingness to use simulation-based education.

**Conclusion:**

The findings of this study indicate that online training is as effective as onsite training in improving simulation facilitator core competency and willingness to use simulation-based education. This suggests that online training could be a viable alternative to onsite training.

## 1 Introduction

Simulation-based training has become an increasingly popular and effective method for educating healthcare professionals across a wide range of disciplines (1–3). Simulation facilitators play a crucial role in the design, implementation, and debriefing of simulation-based learning experiences (4). Consequently, simulation facilitator core competency is critical to the successful delivery of high-quality simulation-based education. This competency encompasses the knowledge, skills, behaviors, and professional attributes required to design, facilitate, and debrief simulation activities effectively, including the ability to create psychologically safe learning environments, integrate theory with clinical practice, promote learner reflection, and demonstrate professional values (5). Qualified and well-trained simulation facilitators are essential to ensure that simulation sessions achieve their intended learning objectives and provide meaningful learning opportunities for participants (6).

As the use of simulation in healthcare education continues to grow (7), there is a need to explore efficient and accessible approaches to train simulation facilitators. Traditional onsite facilitator training programs have been the standard (8), but the COVID-19 pandemic has accelerated the adoption of online modalities across various educational settings (9). Online facilitator training offers several potential advantages, such as increased accessibility, flexibility, and cost-effectiveness, particularly for healthcare organizations with geographically dispersed personnel (10–12). However, online training may also present challenges, including reduced opportunities for hands-on practice, limited face-to-face interaction and peer learning, and potential technical difficulties that may affect participants’ learning experiences (13–16). Despite these potential benefits and limitations, the relative effectiveness of online and onsite simulation facilitator training remains inadequately understood. Determining whether online training can achieve outcomes comparable to those of traditional onsite programs is essential for informing faculty development initiatives, optimizing resource allocation, and guiding institutional and policy decisions, particularly in resource-constrained or geographically dispersed settings.

This study aimed to address this gap by conducting comparative evaluation of online and onsite training programs for simulation facilitators. By examining key outcomes, such as changes in simulation facilitator core competency, the findings will inform the design and implementation of future simulation facilitator training programs that can support high-quality simulation-based education in healthcare.

## 2 Methods

### 2.1 Design

The study employed a quasi-experimental design, with participants assigned to either an online training group or an onsite training group based on their availability and preference. A quasi-experimental design was considered appropriate because random assignment was not feasible within the real-world educational context of this study. This design allowed the effectiveness of the two training modalities to be evaluated while accommodating participants’ logistical constraints and maintaining access to the intervention. The onsite training was conducted in Macau, a region with the highest Gross Domestic Product per capita in China and the fifth highest globally (17). Consequently, participants residing outside Macau would face significantly higher costs to attend the onsite sessions. Furthermore, residents from other Chinese cities are required to obtain visas to travel to Macau, with annual restrictions on the number of visas issued. These logistical constraints rendered random assignment of participants impractical. To facilitate onsite participation among non-Macau residents, the research team’s institution provided supporting documentation for visa applications when required and offered accommodation at subsidized rates.

Due to the inherently distinct nature of the two intervention methods, it was not feasible to blind the participants to their group assignment. However, to maintain objectivity in the analysis, only the data analyst was blinded to the participants’ group allocations. This approach helped to minimize bias during the data analysis phase. Recognizing the potential for confounding arising from the non-randomized group allocation, demographic and professional characteristics, including gender, residency status, years of work experience, and baseline outcome scores, were assessed and subsequently adjusted for in the multivariable analyses. This study followed the guidelines of the Transparent Reporting of Evaluations with Nonrandomized Designs (TREND).

### 2.2 Setting

This research was carried out at the Kiang Wu Nursing College of Macau, an institution with over a century-long tradition in nursing education, from which approximately 70% of Macau’s nursing professionals have graduated. The college is equipped with a high-fidelity nursing laboratory that replicates a hospital’s double-occupancy patient room environment and is outfitted with one-way mirrors. It also includes a SimMan®3G high-fidelity patient simulator. Furthermore, the college houses a simulated maternity and pediatric ward, featuring SuperTory® and SimJunior® high-fidelity simulators for newborns and children. The college has over 10 years of experience in high-fidelity simulation-based training and conducts related research.

### 2.3 Participants

Inclusion Criteria: Participants in this study consisted of physicians or nurses with a minimum of one year of professional experience. Their regular job responsibilities included engagement in the training of nurses, or nursing students.

Exclusion Criteria: Individuals were excluded from the study if they demonstrated a lack of proficiency in the language of training (Chinese) or had an attendance rate below 90% during the training period.

Participants were recruited from universities and hospitals in Macau, Guangdong Province, and Jiangsu Province over a 15-day period, from July 1 to July 15, 2023. Recruitment materials included information about the training program, instructor team, registration procedures, and contact details. To obtain a diverse sample, recruitment was conducted through multiple channels, including institutional intranet or email announcements, direct invitations from the research team, and social media dissemination. Recipients were also encouraged to share the recruitment information with eligible individuals.

Interested individuals were provided with study information describing the study procedures, potential risks and benefits, and the voluntary nature of participation. Participants who provided informed consent completed the registration process by submitting their demographic and professional information, including workplace and years of work experience, and indicating their preferred training format (online or face-to-face). Enrolled participants subsequently received information regarding training arrangements and study procedures.

Sample size was determined using G*Power software (Version 3.1) to ensure adequate statistical power for detecting differences between the online and onsite training groups. For the calculation, an effect size of 0.7 (Cohen’s d) was assumed, with a significance level (*α* error) set at 0.05 (two-tailed) and a desired power (1 - *β* error) of 0.80. A 3:1 allocation ratio (online to onsite) was assumed in the sample size calculation based on the research team’s previous experience with similar training programs, where participant enrollment consistently favored the online format because of its greater accessibility and flexibility. Based on these parameters, the required sample size was calculated to be 52 participants in the online training group and 18 participants in the onsite training group.

### 2.4 Intervention

The training program spanned 3.5 + 0.5 days, amounting to a total of 21 h. Participants engaged in 3.5 days of training, after which they conducted simulation-based education independently in their own institutions. They reconvened in the classroom 2 weeks later for a sharing session. The training was delivered synchronously to both on-site and online participants, utilizing professional live broadcasting services provided by a specialized team equipped with professional video recording and audio collection equipment. Participants and instructors formed social media groups to facilitate ongoing support, allowing participants to seek assistance with any questions at any time.

The training team included three instructors and three teaching assistants. Two instructors, certified as simulation-based education instructor trainers by the National League for Nursing and holding PhDs, led the training on the first 2 days. The third instructor, also a PhD holder with over 15 years of experience in simulation-based education and research, conducted the training on the third day. The teaching assistants, each with a master’s degree and over 5 years of experience in simulation-based education and research, supported the sessions.

This study was informed by Kolb’s Experiential Learning Theory, which served as the pedagogical framework for the design and implementation of the simulation facilitator training program rather than as a framework for evaluating training outcomes. Kolb’s theory highlights four stages of the learning process: Concrete Experience, Reflective Observation, Abstract Conceptualization, and Active Experimentation. These stages constitute a cycle that encourages learners to acquire knowledge and skills through experience and reflection. The training was structured into two phases, as outlined in Table 1.

**Table 1:** Training schedule.

Phase	Specific arrangement		
	Day	Morning (location: one classroom) (all participants in one classroom)	Afternoon (location: two high-fidelity nursing laboratory) (half of the participants (*n* = 11) in a laboratory)
Phase I (Theory Learning + Lab Practice)	Day 1	How to Conduct Simulation-Based Education (3 h)	Simulation-Based Education (From the Student’s Perspective) (3 h) The training instructor facilitated a high-fidelity simulation session, with participants assuming student roles. Among 11 onsite participants, three served as operators and seven as observers, alongside online participants observing remotely. A debriefing session followed.
	Day 2	How to Conduct Debriefing (3 h)	Simulation-Based Education (From the simulation facilitator’s Perspective) (3 h) Participants conducted a high-fidelity simulation session. The 11 onsite participants comprised two facilitators, three operators, and six observers (online participants also observed). This was followed by participant-led debriefing.
	Day 3	How to Conduct Simulation-Based Education Evaluation (3 h)	Simulation-Based Education Evaluation (2 h) A skills examination using a high-fidelity mannequin was conducted by the instructor (as facilitator) and participants. Onsite participants (*n* = 11) included two operators and nine examiners (online participants also served as examiners), followed by debriefing.
	Day 4	Summary and Q&A Session (2 h)	
Phase II (Independent Conduct of Simulation-Based Education + Classroom Sharing)	Day 5 to Day 17	Independent Conduct of Simulation-Based Education During this period, participants can contact the training instructor for assistance at any time via social media.	
	Day 18	Sharing and Q&A Session (2 h)	

In Phase I, encompassing the first 3 days, participants received theoretical instruction in the mornings and engaged in laboratory activities in the afternoons. This structure provided both theoretical knowledge and practical experience, aligning with the Concrete Experience stage. The afternoon laboratory sessions facilitate Reflective Observation and Active Experimentation, enabling participants to validate their acquired knowledge through hands-on activities. These sessions further assisted participants in grasping the theoretical and practical aspects of simulation-based education, aligning with the Abstract Conceptualization stage. Summary sessions at the end of Phase I allowed participants to review and reflect on their learning, aligning with Reflective Observation.

In Phase II, participants independently developed and delivered simulation-based educational sessions, enabling engagement in all four stages of experiential learning: gaining direct teaching experience (Concrete Experience), reflecting on their practices and outcomes (Reflective Observation), conceptualizing teaching theories (Abstract Conceptualization), and applying these concepts to new scenarios (Active Experimentation). Sharing sessions at the end of Phases II facilitated the exchange of insights, corresponding to Abstract Conceptualization. The reflection and observation in the question-and-answer session at the Phase I and Phase II reflected the characteristics of the Reflective Observation.

### 2.5 Outcome measures

The outcome measures were selected based on the Kirkpatrick Model (18), a four-level framework for evaluating training effectiveness. The model was considered particularly appropriate for this study because it provides a comprehensive and hierarchical structure for assessing educational outcomes, encompassing learners’ reactions, knowledge and skill acquisition, behavioral changes, and organizational outcomes. The four outcome measures in this study correspond to Kirkpatrick’s Levels 1 through 3: the willingness to use simulation-based education reflects Level 1 (Reaction), simulation-based education self-efficacy and debriefing skills reflect Level 2 (Learning), and simulation facilitator core competency reflects Level 3 (Behavior).

The primary outcome was simulation facilitator core competency. Secondary outcomes included debriefing skills, simulation-based education self-efficacy, and the willingness to use simulation-based education.

The simulation facilitator core competency was measured using a scale developed by the research team. The development of this scale was based on the Simulation Facilitator Competencies from the Canadian Nurse Educator Institute (19) and the Nurse Educator Core Competency from the World Health Organization (20). An initial pool of items was constructed through expert interviews and literature reviews. This pool underwent two rounds of expert consultation to refine and adjust the items, resulting in a preliminary version of the scale. After testing the preliminary version and conducting psychometric analyses based on participant feedback, the final version of the scale was established. The final scale has 13 items rated on a 5-point Likert scale (1 = Strongly Disagree, 5 = Strongly Agree), with a total score up to 5 (Supplementary Table S1). It achieved a content validity index of 1.0 and a Cronbach’s alpha of 0.95, indicating acceptable reliability and validity.

Debriefing skills were assessed using the self-assessment version of the Debriefing Assessment for Simulation in Healthcare© (DASH) (21). Prior to completing the assessment, participants received an orientation to the DASH tool, including an explanation of its six dimensions and rating criteria, to promote a consistent understanding of the evaluation standards. The self-assessment version was used because the study aimed to examine participants’ perceived debriefing competence and self-reflection regarding their debriefing performance following the training. The DASH comprises six items and uses a 7-point Likert scale (1 = Extremely Ineffective / Detrimental, 7 = Extremely Effective / Outstanding). The total score is the sum of the scores across the six items, with a maximum possible score of 42. The DASH has demonstrated good reliability with the Cronbach’s alpha coefficient for DASH is 0.89 (22).

Simulation-based education self-efficacy was measured using a scale developed by the research team. The development of this scale was grounded in self-efficacy theory. An initial item pool was created through expert interviews and literature reviews, refined it through two rounds of expert consultations, and finalized it after testing and psychometric analysis. The final scale consists of 6 items rated on a 5-point Likert scale (1 = Not at all Confident, 5 = Extremely Confident), with a total score up to 5 (Supplementary Table S2). It achieved a content validity index of 1.0 and a Cronbach’s alpha of 0.94, indicating acceptable reliability and validity.

The willingness to use simulation-based education was measured using a scale developed by the research team. This scale includes three items to assess participants’ willingness: “If needed, I am willing to try conducting simulation-based education,” “I am willing to recommend others to conduct simulation-based education,” and “In the future, I will conduct more simulation-based education.” Responses were on a 5-point Likert scale from strongly disagree to strongly agree, with a total score up to 5. Expert consultation confirmed content validity with an index of 1.0, and testing with 215 participants showed a Cronbach’s alpha of 0.95, indicating acceptable reliability and validity.

### 2.6 Data collection

**Figure 1:**
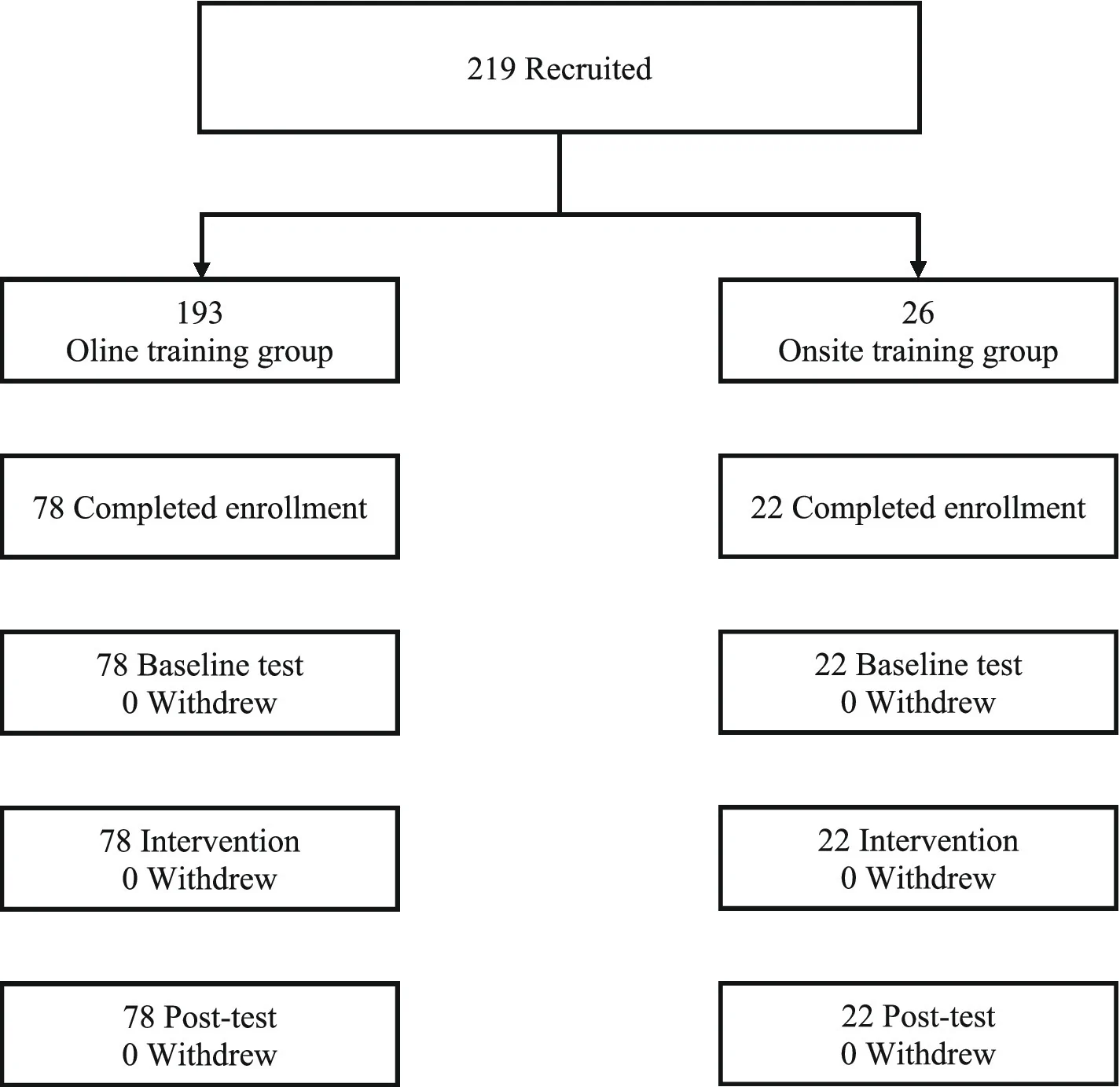
Study subject disposition flow chart.

The study flow chart is shown in Figure 1. Data were collected at two points: baseline (within a week before the intervention) and immediately post-intervention (within a week following the intervention). Baseline data included demographics, simulation facilitator core competency, debriefing skills, simulation-based education self-efficacy, and the willingness to use simulation-based education. Post-intervention assessments covered the same areas except demographics. Participants completed the survey independently via an electronic questionnaire accessed through a QR code.

### 2.7 Statistical analysis

Given that one group had fewer than 30 participants and the data did not meet the criteria for normal distribution after a normality test, non-parametric statistical methods were employed for the analysis. Descriptive statistics were used to describe the participant characteristics, baseline characteristics, and post-intervention characteristics. The median, first quartile and third quartile were used to express continuous variables, while frequencies and percentages were employed to present categorical variables. The baseline characteristics of the two groups were compared using Mann–Whitney U test. Pairwise comparisons were performed using the Wilcoxon signed rank test. Changes in the outcomes were calculated as the percentage change from the baseline. The changes among the two groups were compared using multiple linear regression. Analyses were performed using jamovi software version 2.3.28. Results were considered significant when *p* < 0.05.

## 3 Results

### 3.1 Participants

Of the 219 individuals who initially expressed interest in participating, 100 enrolled in and completed the intervention and post-intervention assessments, including 78 participants in the online training group and 22 participants in the onsite training group (Figure 1). Baseline characteristics were generally comparable between the online and onsite training groups. No significant between-group differences were observed in gender distribution, residency status, years of work experience, simulation facilitator core competency, or simulation-based education self-efficacy. However, the online training group exhibited significantly lower debriefing skills and willingness to use simulations-based education compared to the onsite training group (Table 2).

**Table 2:** Participant demographics and baseline characteristics.

Variable	Online training group (*n* = 78)	Onsite training group (*n* = 22)	U/χ^2^	*p*
Gender ^a^	72 (6)	21 (1)	0.261	0.609
Resident	47 (31)	17 (5)	2.16	0.142
Years of work experience ^c^	14.0 (9.0–20.0)	17.0 (9.8–23.5)	755	0.393
Simulation facilitator core competency ^c^	3.8 (3.4–4.0)	3.9 (3.7–4.1)	659	0.098
Debriefing skills ^c^	30.0 (25.3–33.8)	31.0 (30.0–35.8)	607	0.036
Simulation-based education self-efficacy ^c^	3.3 (3.0–4.0)	3.5 (3.2–4.0)	823	0.769
Willingness to use simulation-based education ^c^	4.0 (4.0–4.7)	4.3 (4.0–5.0)	588	0.017

^a^The data are presented as male (female) and are the outcome of chi-square test. ^b^The data are presented as non-Macau resident (Macau resident) and are the outcome of chi-square test. ^c^The data are presented as median (first quartile - third quartile) and are the outcome of Mann–Whitney U test.

### 3.2 Primary outcomes

Following the intervention, both the online and onsite training groups exhibited significant improvements in simulation facilitator core competency. In the online group, the median score increased from 3.8 at baseline to 4.0 post-intervention (*p* < 0.001). Similarly, the onsite group showed an increase from 3.9 to 4.0 (*p* = 0.012) (Table 3). However, after adjustment for potential confounders, the percentage change from baseline did not differ significantly between the online and onsite groups (*β* = 0.044, *p* = 0.811) (Table 4).

**Table 3:** Results of the evaluation of the effects before and after the intervention.

Variable	Online training group (*n* = 78)				Onsite training group (*n* = 22)			
	Baseline	Post-intervention	W	*p*	Baseline	Post-intervention	W	*p*
Simulation facilitator core competency	3.8 (3.4–4.0)	4.0 (3.9–4.2)	324	<0.001	3.9 (3.7–4.1)	4.0 (3.8–4.4)	43.0	0.012
Debriefing skills	30.0 (25.3–33.8)	30.5 (30.0–36.0)	228	<0.001	31.0 (30.0–35.8)	33.0 (29.3–36.0)	103.5	0.689
Simulation-based education self-efficacy	3.3 (3.0–4.0)	3.8 (3.2–4.0)	687	0.018	3.5 (3.2–4.0)	3.8 (2.8–4.1)	55.5	0.535
Willingness to use simulation-based education	4.0 (4.0–4.7)	4.0 (4.0–4.0)	621	0.030	4.3 (4.0–5.0)	4.0 (4.0–4.0)	78.0	0.001

The data are presented as median (first quartile - third quartile).

**Table 4:** Results of the evaluation of the percentage change from the baseline.

Variable	B	SE	*t*	*p*	Standardized β	95% CI
Simulation facilitator core competency						
Group (Reference = Online)	0.007	0.028	0.240	0.811	0.044	−0.318–0.406
Gender (Reference = Female)	0.007	0.046	0.153	0.879	0.046	−0.545–0.636
Resident (Reference = Macau resident)	−0.009	0.025	−0.353	0.725	−0.056	−0.371–0.259
Baseline	−0.212	0.024	−8.831	<0.001	−0.676	−0.828–-0.524
Years of work experience	−0.001	0.002	−0.973	0.333	−0.074	−0.225–0.077
R^2^ = 0.484						
Debriefing skills						
Group (Reference = Online)	0.016	0.085	0.189	0.851	0.038	−0.358–0.433
Gender (Reference = Female)	−0.017	0.137	−0.126	0.900	−0.041	−0.680–0.598
Resident (Reference = Macau resident)	0.151	0.074	2.032	0.045	0.355	0.008–0.703
Baseline	−0.053	0.007	−7.627	<0.001	−0.642	−0.809–-0.475
Years of work experience	−0.003	0.004	−0.653	0.516	−0.053	−0.214–0.108
R^2^ = 0.393						
Simulation-based education self-efficacy						
Group (Reference = Online)	−0.038	0.053	−0.719	0.474	−0.161	−0.604–0.283
Gender (Reference = Female)	−0.049	0.088	−0.552	0.582	−0.203	−0.931–0.526
Resident (Reference = Macau resident)	−0.030	0.047	−0.629	0.531	−0.123	−0.513–0.266
baseline	−0.174	0.036	−4.793	<0.001	−0.443	−0.626–-0.259
Years of work experience	−0.001	0.003	−0.376	0.708	−0.035	−0.219–0.149
R^2^ = 0.212						
Willingness to use simulation-based education						
Group (Reference = Online)	−0.001	0.006	−0.223	0.824	−0.010	−0.102–0.081
Gender (Reference = Female)	0.005	0.010	0.435	0.664	0.032	−0.115–0.179
Resident (Reference = Macau resident)	−0.011	0.006	−1.919	0.058	−0.079	−0.160–0.003
Baseline	−0.235	0.005	−49.018	<0.001	−0.971	−1.010–-0.931
Years of work experience	0.000	0.000	0.953	0.343	0.018	−0.019–0.055
R^2^ = 0.968						

### 3.3 Secondary outcomes

Within the online training group, there were significant enhancements in debriefing skills (median: 30.0 vs. 30.5, *p* < 0.001), simulation-based education self-efficacy (3.3 vs. 3.8, *p* = 0.018), and the willingness to use simulation-based education (4.0 vs. 4.0, *p* = 0.030) (Table 3). Conversely, the onsite training group did not show significant improvements in debriefing skills (31.0 vs. 33.0, *p* = 0.689) and simulation-based education self-efficacy (3.5 vs. 3.8, *p* = 0.535) (Table 3). Notably, the onsite group experienced a significant decline in their willingness to use simulation-based education (4.3 vs. 4.0, *p* = 0.001) (Table 3). When comparing the two groups, there were no statistically significant differences in the percentage change from baseline for debriefing skills (*β* = 0.038, *p* = 0.851), simulation-based education self-efficacy (β = −0.161, *p* = 0.474), and the willingness to use simulation-based education (β = −0.010, *p* = 0.824) (Table 4).

## 4 Discussion

To our knowledge, few studies have directly compared the effects of online and onsite training for simulation facilitators, and the present study adds to the emerging evidence in this area. The results encompassed the contributions of both online and on-site training to four aspects of simulation facilitators: facilitator core competency, debriefing skills, simulation-based education self-efficacy, and the willingness to use simulation-based education.

This study demonstrated that both online and onsite training effectively enhance the core competence of simulation facilitators, with no significant difference in the percentage change from baseline competency between the two groups. The findings indicate that both training modalities can achieve comparable outcomes in developing simulation facilitator core competence. These results align with recent studies (23, 24), such as one that compared four training modes for healthcare professionals and found no statistically significant differences in learning outcomes among onsite, videoconference, online, and blended formats (23). This suggests that when training is effectively designed and delivered, the mode of delivery may not substantially influence learning outcomes (25). This challenges the assumption that the face-to-face interactions inherent in onsite training are more beneficial for skill development than online formats. This finding is particularly pertinent given the increasing trend toward online education and training, where accessibility and flexibility offer significant advantages (26). However, it is important to consider the broader implications of these findings. While both training formats were effective in enhancing core competency, the mechanisms might differ. Onsite training might offer more immediate feedback and hands-on practice, while online training might provide a more self-paced learning environment (27, 28). The results also raise questions about the sustainability of the competency gained. Future research could explore the long-term impact of online versus onsite training on simulation facilitator core competency, including how the core competency is applied in practice and whether there are differences in retention rates between the two formats.

The findings of this study indicated that both online and onsite training were effective in enhancing the debriefing skills among participants. Debriefing is a critical component of simulation-based education (29). Debriefing facilitates a structured reflection on the simulation experience, allowing learners to critically assess their performance, identify areas for improvement, and reinforce knowledge and skills (30, 31). This reflective process is crucial for deep learning and helps in translating simulation experiences into real-world clinical practice (32, 33). The improvement observed in both groups highlights the potential of both training modalities to support the development of this essential skill. However, the findings of this study revealed no significant difference between the two groups regarding the percentage change from baseline, suggesting that the effect of online training is comparable to that of onsite training in developing participants’ debriefing skills. This aligns with the outcomes of most studies, with a meta-analysis showing that out of 69 included studies, 46 demonstrated equivalent effects between online and onsite training (34). The effectiveness of online training is influenced by factors such as course design, teacher capabilities, and student attributes. An effective online training requires: (1) a course with a clear structure, authentic and inclusive learning activities, and high-quality online interaction; (2) teachers with online teaching competencies; and (3) students with high self-regulation skills and sufficient digital literacy (35). The team involved in the development of the training curriculum for this study all possess over 15 years of experience in simulation-based education or are certified simulation-based education instructor trainers, and they all hold doctoral degrees. The teaching team also has over 5 years of experience in online teaching. The live broadcasting equipment and related professional support personnel required for the online training were provided by a professional company. The participants in this study are all professionals, ensuring they have adequate self-regulation skills and digital literacy. All these guarantee the effectiveness of the online training. It is important to acknowledge that while online training can be equivalent in effectiveness, it may not be suitable for all types of skills development. Certain skills require learning through hands-on practice and interpersonal interaction, aspects that are challenging to fully replicate in online environments (36). Future research should explore the comparative effectiveness of online and on-site training in developing other essential skills for simulation facilitators.

The results of this study revealed differing patterns of change in willingness to use simulation-based education between the two training modalities. Participants in the online training group demonstrated a significant increase in willingness to use simulation-based education, whereas those in the onsite training group showed a significant decrease. The decline observed in the onsite training group warrants further investigation. Several factors could have contributed to this outcome. The onsite training format, while traditionally valued for its hands-on approach and direct interaction with instructors (37), may have posed significant logistical challenges for participants, such as travel time, scheduling conflicts, and the need to balance training with other professional responsibilities (38–40). These challenges can create additional stress and fatigue, potentially leading to a less favorable perception of the training experience (41). Moreover, the intensive nature of onsite training sessions may have resulted in cognitive overload for some participants (42). The requirement to absorb a large volume of information in a short period can be overwhelming, reducing the overall effectiveness of the training and diminishing participants’ motivation to apply what they have learned (43). The lack of flexibility in onsite training may also limit opportunities for individualized learning and reflection, further contributing to the observed decline in willingness to use simulation-based education. However, despite these within-group changes, the adjusted analyses demonstrated no statistically significant between-group differences in the percentage change from baseline for willingness to use simulation-based education. Therefore, the observed changes in willingness within each group should be interpreted with caution and should not be considered evidence of the superiority of one training modality over the other. The absence of significant between-group differences has important implications for faculty development in simulation-based education. Online training may represent a viable alternative to traditional onsite training, particularly when geographical, financial, or scheduling barriers limit access to in-person programs. At the same time, onsite training continues to offer opportunities for direct interaction and experiential learning. Institutions may therefore consider selecting training modalities based on learner needs, available resources, and contextual factors rather than assuming inherent differences in effectiveness between online and onsite approaches.

## 5 Limitation

Several limitations must be acknowledged that could influence the interpretation and generalizability of our findings. Firstly, the sample size, particularly in the onsite training group, was relatively small, potentially affecting the study’s power to detect statistically significant differences and limiting the generalizability of the results. In addition, the substantial imbalance in group sizes between the online (*n* = 78) and onsite (*n* = 22) training groups may have affected the precision of between-group comparisons. Secondly, the quasi-experimental design without randomization may have introduced biases, including selection bias, as participants were assigned to groups based on their availability and preferences rather than through random allocation. Consequently, despite statistical adjustment for measured covariates, the influence of unmeasured confounding variables cannot be excluded. Moreover, data collection relied on self-report questionnaires, which are susceptible to response bias and may not accurately capture participants’ true attitudes and behaviors. Lastly, the study focused on immediate post-intervention outcomes without assessing long-term retention or the impact on actual teaching practices, which is a critical area for future research to explore. Considering these limitations is essential for a more nuanced understanding of our results and for planning more robust studies in the future.

## 6 Conclusion

This study found that both online and onsite simulation facilitator training were associated with improvements in simulation facilitator core competency and willingness to use simulation-based education, with no significant differences observed between the two training modalities, suggesting comparable effectiveness. However, given the study’s quasi-experimental design, non-randomized group allocation, unequal group sizes, and limited sample size, the findings should be interpreted with caution and cannot be generalized beyond the study sample. Further studies involving larger and more diverse populations, together with rigorous experimental designs, are warranted to establish the effectiveness of faculty development programs for simulation facilitators and to determine their long-term educational impact.

## Data Availability

The raw data supporting the conclusions of this article will be made available by the authors, without undue reservation.
