# Supplementary material for: Comparing the effectiveness of online and onsite training for simulation facilitator: a quasi-experiment
Source: Front Med (Lausanne). 2026 Aug 13;13:1854386. doi: 10.3389/fmed.2026.1854386 (PMC13518188; doi:10.3389/fmed.2026.1854386)
Supplement: Supplementary file 1 [file Table_1.DOCX]

Supplemental material

Table S1 Simulation facilitator core competency scale

The scale comprises 12 statements related to your simulation-based education. Please indicate your level of agreement or disagreement with each statement by circling the corresponding number on the scale provided below. Please provide one answer for each statement.

|  | 1 Strongly Disagree | 2 | 3 | 4 | 5 Strongly Agree |
| --- | --- | --- | --- | --- | --- |
| 1. I possess theoretical foundation in simulation-based education. |  |  |  |  |  |
| 2. I acquire skills for conducting simulation-based education. |  |  |  |  |  |
| 3. I apply theory-based frameworks to guide practice. |  |  |  |  |  |
| 4. I design up-to-date simulation-based education activities. |  |  |  |  |  |
| 5. I select appropriate assessment methods. |  |  |  |  |  |
| 6. I establish clear learning/teaching objectives based on student levels. |  |  |  |  |  |
| 7. I utilize teaching techniques to stimulate student interest. |  |  |  |  |  |
| 8. I create positive simulation environment for learning. |  |  |  |  |  |
| 9. I perform learning assessments based on objectives. |  |  |  |  |  |
| 10. I provide timely feedback to students on assessments. |  |  |  |  |  |
| 11. I embrace constructive feedback. |  |  |  |  |  |
| 12. I promote collaboration among colleagues. |  |  |  |  |  |

Table S2 Simulation-based education self-efficacy scale

When faced with the following situations, please indicate your level of confidence in conducting simulation-based education:

|  | Not at all confident | Slightly confident | Somewhat confident | Quite confident | Extremely confident |
| --- | --- | --- | --- | --- | --- |
| 1. When there is no simulation teaching team available. |  |  |  |  |  |
| 2. When the trainees come from different professional backgrounds. |  |  |  |  |  |
| 3. When previous simulation teaching outcomes were not satisfactory. |  |  |  |  |  |
| 4. When you lack relevant clinical experience. |  |  |  |  |  |
| 5. When you have insufficient experience in simulation-based education. |  |  |  |  |  |
| 6. When there are too many trainees. |  |  |  |  |  |
